# COMMENTARY ON: “EFFECTS OF MOTOR IMAGERY-BASED NEUROFEEDBACK TRAINING AFTER BILATERAL REPETITIVE TRANSCRANIAL MAGNETIC STIMULATION ON POST-STROKE UPPER LIMB MOTOR FUNCTION: AN EXPLORATORY CROSSOVER CLINICAL TRIAL”

**DOI:** 10.2340/jrm.v56.41133

**Published:** 2024-08-28

**Authors:** Anjali RAGHUWANSHI, Saliha RAFAT, Adarsh KUMAR SRIVASTAV

**Affiliations:** Department of Physiotherapy, School of Health Sciences, Chhatrapati Shahu Ji Maharaj University, Kalyanpur, Kanpur-208024, Uttar Pradesh, India

*To the Editor,*

We have read the article by Francisco Jose Sanchez-Cuesta et al. (1), with great interest. The introduction is well explained and written. Also, the author had well explained the treatment effect of both rTMS and NFB. We gathered valuable information and wish to congratulate the authors for this successful clinical trial. However, there are some concerns that need to be addressed.

The sample size of the study was well explained in detail related to previous studies. But the subjects were taken from one centre, so identified risk factors may be exclusive to that single centre. This methodological choice may weaken the generalizability of the study findings (2). In the clinical trial, the title is “Transcranial Magnetic Stimulation and Mental Representation Techniques for the Treatment of Stroke Patients”, but in the manuscript it is “Effects of Motor Imagery-Based Neurofeedback Training After Bilateral Repetitive Transcranial Magnetic Stimulation on Post-Stroke Upper Limb Motor Function: An Exploratory Crossover Clinical Trial”.

While the AB/BA crossover design employed in this study mitigates individual differences and potentially reduces bias (3), the lack of correction for the multiple comparisons and potential learning effects from repeated assessments could compromise the validity of the findings. This broad problem-focused approach, prioritizing a generalizable effect, may neglect the core issue of treatment efficacy for the distinct stroke population (subacute vs chronic). There is a lack of specificity subgroups. A more focused investigation with a clear evaluation of treatment efficacy in subacute and chronic stroke populations is warranted. The potential for bias extends beyond the choice of study design. Selection bias is the concern, as the inclusion criteria are not specific enough to represent the sample. Blinding bias could also occur if either researchers or participants were aware of the intervention assignment, potentially influencing behaviour and skewing the results. Finally, performance bias cannot be ruled out, as the factors not directly related to the intervention could have influenced participant performance (4).

rTMS is a very safe and non-invasive technique used to treat various psychiatric and neurological disorders by stimulating specific brain regions with magnetic fields. rTMS can increase or decrease cortical excitability, which manages the symptoms of the aforementioned conditions (5). While in the study intervention it was not clear if the rTMS was given for 10 consecutive days in therapy group A, then why in therapy group B MI-NFB was it given non-consecutively for 12 days and also why were the first 6 MI-NFB sessions carried out after rTMS and the last 6 sessions, without rTMS?

The results of the study indicate that the combined therapy can be promising treatment for stroke patients in both the subacute and chronic phases. Clinicians may consider incorporating motor imagery-based neurofeedback training and repetitive transcranial magnetic stimulation into rehabilitation programmes for stroke survivors. However, the study did not assess the persistence of intervention effects beyond 1 month. Future research could explore the long-term effects of the combined therapy to better understand its sustainability and impact on functional outcomes.

In addition to our concerns, the study does not represent the size and clinical importance of the effect of MI-NFB or MI-NFB after rTMS. Statistical significance is not the same as clinical significance. Thus, further statistical analysis was performed to obtain the effect size and power of the study by using G*Power because effect size and power are crucial for designing a strong crossover trial. They help researchers to identify the practical significance of treatment effects, plan for an adequate sample size, and avoid both false negatives and false positives in the analysis (6, 7). Multiple formulas were used to compute the effect size, depending on the analysis and type, such as (8):

*1. Cohen’s d*

Cohen’s d is used to measure the effect size for the difference between 2 means.


$$d=\frac{M_{1}-M_{2}}{\sqrt{\frac{s_{1}^{2}+s_{2}^{2}}{2}}}$$

where M1 and M2 are the means of the 2 groups and s1 and s2 are the standard deviations of the two groups (9).

*2. Hedges’ g*

This is a variation of Cohen’s d, but with a correction factor applied for small sample sizes.


$$\mathrm{Hedges}’\;g=\left(\frac{M1-M2}{\mathrm{SD}*\mathrm{pooled}}\right)$$

where M1 and M2 are differences in mean and SD* _pooled_ is pooled and weighted standard deviation (10).

Hedges’ g and Cohen’s d are interpreted similarly. For analysing outcomes, Cohen recommended applying the following general rule of thumb: small effect: < 0.2, medium effect: 0.5, large effect: 0.8) (11).

The Fugl–Meyer Assessment of the Upper Limb (FMA-UL) showed a clinically significant effect size for group AB (Cohen’s d = 0.75; power = 0.98) and group BA (Cohen’s d = 1.55; power = 1.00). The Hand Grip test revealed a small effect size for group AB (Cohen’s d = 0.23, power = 0.30) and a medium effect size for group BA (Cohen’s d = 0.54; power = 0.84). The Nottingham Sensory Assessment (NSA) Total Score (TS) showed a medium effect size for group AB (Cohen’s d = 0.47; power = 0.75) and a large effect size for group BA (Cohen’s d = 1.00; power = 0.99). The NSA Kinesthesia Score (KS) had a large effect size for group AB (Cohen’s d = 1.2; power = 0.99) and a small effect size for group BA (Cohen’s d = 0.41; power = 0.65). The NSA Stereognosis Score (S) revealed a small effect size for both group AB (Cohen’s d = 0.10; power = 0.12) and group BA (Cohen’s d = 0.09; power = 0.11). The Nine-Hole Peg Test (9-HPT) showed a medium effect size for group AB (Cohen’s d = 0.45; power = 0.72) and a large effect size for group BA (Cohen’s d = 0.70; power = 0.96). The Action Research Arm Test (ARAT) had a large effect size for both group AB (Cohen’s d = 1.01; power = 0.99) and group BA (Cohen’s d = 1.21; power = 0.99). Finally, the Finger Tapping Test (FTT) revealed a small effect size for group AB (Cohen’s d = 0.10; power = 0.12) and a medium effect size for group BA (Cohen’s d = 0.51; power = 0.81).

This study’s findings suggest that combining motor imagery-based neurofeedback training with repetitive transcranial magnetic stimulation can improve upper limb affected patients. This has important implications for enhancing rehabilitation strategies for stroke survivors. The study adds to the existing literature by demonstrating the potential benefits of combining these 2 interventions for stroke rehabilitation. The findings support the efficacy of the combined approach in improving motor outcomes and sensory function. The study provides valuable information, but the above-mentioned points need to be considered for clinical interpretation. We would be interested in the authors’ thoughts on these comments.

## References

[1] Sánchez Cuesta FJ, González-Zamorano Y, Moreno-Verdú M, Vourvopoulos A, Serrano IJ, Del Castillo-Sobrino MD, et al. Effects of motor imagery-based neurofeedback training after bilateral repetitive transcranial magnetic stimulation on post-stroke upper limb motor function: an exploratory crossover clinical trial. J Rehabil Med 2024; 56: jrm18253. 10.2340/jrm.v56.18253 38450442 PMC10938141

[2] Wang X, Cheng Z. Cross-sectional studies: strengths, weaknesses, and recommendations. Chest 2020; 158: S65–S71. 10.1016/j.chest.2020.03.012 32658654

[3] Yin RK. Case study research and applications: design and methods. Thousand Oaks, CA: Sage; 2018.

[4] Pannucci CJ, Wilkins EG. Identifying and avoiding bias in research. Plast Reconstr Surg 2010; 126: 619–625. 10.1097/PRS.0b013e3181de24bc 20679844 PMC2917255

[5] Gersner R, Kravetz E, Feil J, Pell G, Zangen A. Long-term effects of repetitive transcranial magnetic stimulation on markers for neuroplasticity: differential outcomes in anesthetized and awake animals. J Neursci 2011; 31: 7521–7526. 10.1523/JNEUROSCI.6751-10.2011 PMC6622610 21593336

[6] Jones B, Kenward MG. Design and analysis of cross-over trials. London: Chapman & Hall/CRC; 2003. 10.1201/9781420036091

[7] Cohen J. Statistical power analysis for the behavioral sciences. London: Routledge; 2013. 10.4324/9780203771587

[8] Durlak JA. How to select, calculate, and interpret effect sizes. J Pediatr Psychol 2009; 34: 917–928. 10.1093/jpepsy/jsp004 19223279

[9] Cohen J. Statistical power analysis for the behavioral sciences [Internet]. 2013 May 13 [cited 2024 Jul 5]; Available from: https://www.taylorfrancis.com/books/mono/10.4324/9780203771587/statistical-power-analysis-behavioral-sciences-jacob-cohen https://doi.org/10.4324/9780203771587

[10] Hedges LV, Hedges VL. Distribution theory for Glass’s estimator of effect size and related estimators. J Educ Behavi Stat 1981; 6: 107–128. 10.3102/10769986006002107

[11] Nakagawa S, reviews ICB, 2007 undefined. Effect size, confidence interval and statistical significance: a practical guide for biologists. Wiley Online Library 2007; 82: 591–605. 10.1111/j.1469-185X.2007.00027.x 17944619

