# Supplementary material for: COMMENTARY ON: “EFFECTS OF MOTOR IMAGERY-BASED NEUROFEEDBACK TRAINING AFTER BILATERAL REPETITIVE TRANSCRANIAL MAGNETIC STIMULATION ON POST-STROKE UPPER LIMB MOTOR FUNCTION: AN EXPLORATORY CROSSOVER CLINICAL TRIAL”
Source: J Rehabil Med. 2024 Aug 28;56:41133. doi: 10.2340/jrm.v56.41133 (PMC11367675; doi:10.2340/jrm.v56.41133)
Supplement: Related texts

# Reply: RESPONSE TO THE COMMENTARY ON: “EFFECTS OF MOTOR IMAGERY-BASED NEUROFEEDBACK TRAINING AFTER BILATERAL REPETITIVE TRANSCRANIAL MAGNETIC STIMULATION ON POST-STROKE UPPER LIMB MOTOR FUNCTION: AN EXPLORATORY CROSSOVER CLINICAL TRIAL”

**Authors:** Francisco Jose SANCHEZ-CUESTA

**Affiliations:** Faculty of Experimental Sciences, Francisco de Vitoria University, Pozuelo de Alarcón, Spain

*Dear Editor,*

We sincerely thank Dr Anjali Raghuwanshi, Dr Saliha Rafat, and Dr Adarsh Kumar Srivastav for their thoughtful commentary on our recently published study. We appreciate their interest in our work and the constructive feedback provided. Herein, we address the concerns raised and provide further clarification on specific points.

*1. Sample size and single-centre study:* While we acknowledge that conducting a study at a single centre may limit the generalizability of our findings, this approach allowed us to maintain a high degree of control over the study environment and intervention protocols. Future research should indeed aim to include multiple centres to enhance the generalizability of the results.

*2. Study title discrepancy:* We appreciate the observation regarding the discrepancy between the trial registration title and the manuscript title. The registered title, “Transcranial Magnetic Stimulation and Mental Representation Techniques for the Treatment of Stroke Patients”, broadly describes the study’s scope. The manuscript title was refined to reflect the specific focus on motor imagery-based neurofeedback (MI-NFB) and repetitive transcranial magnetic stimulation (rTMS).

*3. Crossover design and potential biases:* We selected the AB/BA crossover design to mitigate individual differences and reduce bias. We agree that this design might introduce potential biases such as learning effects. However, the inclusion of a washout period aimed to minimize these effects. The suggestion to address multiple comparisons and blinding in future studies is well taken, and we will incorporate these considerations in subsequent research.

*4. Intervention protocol clarification:* Regarding the protocols of both therapies, we strictly adhered to the parameters as originally published by their respective authors, ensuring no modifications were made. First, Therapy A consisted of 10 sessions of bilateral rTMS. In contrast, Therapy B was a combination therapy, which included the same 10 sessions of bilateral rTMS from Therapy A, plus an additional 12 sessions of IM-NFB training, conducted at a frequency of 3 sessions per week over 4 weeks. Consequently, in the combined therapy, the initial 6 IM-NFB training sessions were immediately preceded by bilateral rTMS, while the subsequent 6 sessions were not.

*5. Long-term effects and clinical significance:* We agree on the importance of assessing long-term effects and the clinical significance of our findings. The current study provided preliminary evidence of the combined therapy’s efficacy. We plan to conduct follow-up studies to explore the long-term sustainability of these interventions and to further analyse clinical outcomes.

*6. Statistical analysis and effect sizes:* The commentary emphasizes the necessity of calculating effect sizes and statistical power. Our study included these analyses to provide a comprehensive understanding of the intervention’s impact. We acknowledge the critical role of these metrics in interpreting the clinical relevance of our findings and will ensure they are prominently reported in future publications.

*Conclusion:* We are grateful for the detailed review and valuable suggestions provided by Dr Raghuwanshi, Dr Rafat, and Dr Srivastav. Their insights will undoubtedly enhance the quality of future research in this area. We remain committed to advancing the understanding and application of neuromodulation techniques in stroke rehabilitation.

Thank you once again for the opportunity to address these comments. We look forward to continuing the dialogue and contributing to the field of neurorehabilitation.
